# Prevalence and correlates of frailty in older hypertensive outpatients according to different tools: the HYPER-FRAIL pilot study

**DOI:** 10.1097/HJH.0000000000003559

**Published:** 2023-10-03

**Authors:** Giulia Rivasi, Ludovica Ceolin, Giada Turrin, Virginia Tortu’, Maria Flora D’Andria, Giuseppe Dario Testa, Sara Montali, Francesco Tonarelli, Enrico Brunetti, Mario Bo, Roman Romero-Ortuno, Enrico Mossello, Andrea Ungar

**Affiliations:** a Division of Geriatric and Intensive Care Medicine, Careggi Hospital and University of Florence, Florence; b Section of Geriatrics, Department of Medical Sciences, University of Turin, Città della Salute e della Scienza, Molinette, Turin, Italy; c Discipline of Medical Gerontology and Falls and Syncope Unit, Mercer's Institute for Successful Ageing, St. James's Hospital, Dublin, Ireland

**Keywords:** blood pressure, comorbidity, depression, hypertension, physical performance

## Abstract

**Objectives:**

To date, few studies have investigated frailty in hypertensive individuals. This study aimed at identifying the prevalence of frailty in a sample of hypertensive older outpatients using six different identification tools. Clinical correlates of frailty and agreement between different frailty definitions were also investigated.

**Methods:**

The HYPER-FRAIL pilot study recruited hypertensive patients aged at least 75 years from two geriatric outpatient clinics of Careggi Hospital, Florence, Italy. Four frailty scales [Fried Frailty Phenotype, Frailty Index, Clinical Frailty Scale (CFS), Frailty Postal Score] and two physical performance tests [Short Physical Performance Battery (SPPB) and usual gait speed] were applied. The Cohen's kappa coefficient was calculated to assess agreement between measures. Multiple logistic regression was used to identify clinical features independently associated with frailty.

**Results:**

Among 121 participants (mean age 81, 60% women), frailty prevalence varied between 33 and 50% according to the tool used. Moderate agreement was observed between Fried Frailty Phenotype, Frailty Index and SPPB, and between Frailty Index and CFS. Agreement was minimal or weak between the remaining measures (K < 0.60). Use of walking aids and depressive symptoms were independently associated with frailty, regardless of the definition used. Frailty correlates also included dementia, disability and comorbidity burden, but not office and 24-h blood pressure values.

**Conclusion:**

Frailty is highly prevalent among older hypertensive outpatients, but agreement between different frailty tools was moderate-to-weak. Longitudinal studies are needed to assess the prognostic role of different frailty tools and their clinical utility in the choice of antihypertensive treatment.

## Graphical abstract

**Figure:**
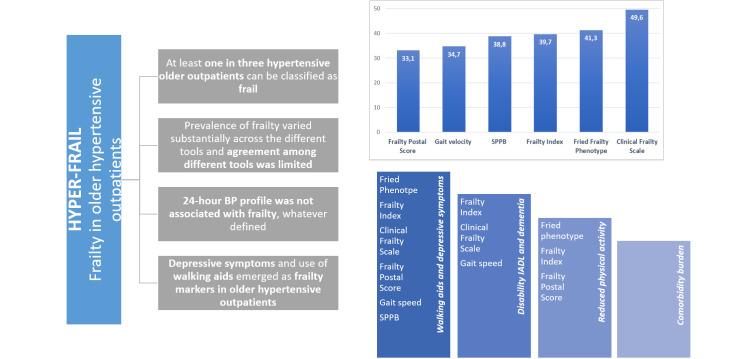

## INTRODUCTION

Frailty is a geriatric syndrome characterized by a decline in older individuals’ physiological functional reserve, resulting in increased vulnerability to external stressors [1]. Frailty thus reduces homeostatic capacity, increasing the risk of adverse health outcomes such as disability, hospitalization, institutionalization and mortality [2,3].

In recent years, frailty has gained a central role in the cardiovascular literature, due to increasing evidence of its high prevalence and substantial prognostic influence in older patients with cardiovascular diseases [4,5]. Moreover, a growing body of research indicates that incorporation of frailty in patients’ assessment and risk stratification may help distinguish older individuals who may benefit from therapeutic interventions in the context of cardiovascular medicine [6,7].

Hypertension is highly prevalent among older people living with frailty, with seven out of 10 frail adults being hypertensive [8,9]. The absolute benefits of antihypertensive treatment are potentially higher in these patients, due to the high cardiovascular risk associated with frailty [10,11]. Yet, treatment benefits must be balanced against the potential risks deriving from increased vulnerability to treatment-related adverse events such as hypotension and falls [12,13]. Moreover, frail individuals have lower life expectancy, due to competing conditions, which play a more relevant role in patients’ prognosis as compared to hypertension [14]. Limited life expectancy and higher risk of treatment-related adverse events may downgrade the prognostic impact of hypertension in frail older adults, thus modifying the risk/benefit ratio of blood pressure (BP) lowering. Indeed, in recent years, several population-based cohort studies have provided evidence of an attenuated or even inverted relationship between BP and mortality in old age, especially in the presence of frailty [15–20]. Moreover, observational data have shown worsening disability and accelerated cognitive decline in older patients with lower BP values [21,22], thus raising concerns about the safety and the extent of BP lowering in frailer subgroups.

Despite these premises, frailty has been poorly investigated in hypertensive patients [23,24] and only two randomized controlled trials have included frailty identification while assessing the prognostic impact of antihypertensive treatment in geriatric patients [25,26]. A multitude of frailty instruments exists in the literature [27], but as different tools capture different types of patients [28], it is still unknown as to which tool best informs clinical decisions on BP management in older patients. These instruments might be especially useful in nongeriatric settings, wherein a comprehensive geriatric assessment is not routinely conducted.

The HYPERtension and FRAILty in Older Adults (HYPER-FRAIL) pilot study aimed to assess the prevalence of frailty in a sample of hypertensive older outpatients using six of the most widely used frailty identification tools. Moreover, we investigated agreement between different frailty definitions and the association of frailty with clinical variables, including 24-h BP profile.

## MATERIALS AND METHODS

The prospective observational HYPER-FRAIL pilot study was conducted at the Hypertension Clinic and Alzheimer's Dementia Evaluation Unit of the Division of Geriatric and Intensive Care Medicine, Careggi University Hospital, Florence, Italy. Patients were screened twice a week between December 2019 and July 2021 and hypertensive patients aged 75 years or older treated with antihypertensive medications were enrolled consecutively. Exclusion criteria included terminal illness (life expectancy <6 months) and refusal of participation by patient and/or his/her legally authorized representative.

All participants underwent a Comprehensive Geriatric Assessment (CGA), including full medical history, comorbidity and medications, physical examination, physical performance, functional and cognitive status and depressive symptoms. Moreover, an office BP measurement and a 24-h ambulatory blood pressure monitoring (ABPM) were performed, and mean home BP values were recorded if a home BP diary was available.

### Frailty Identification

Frailty was identified using four frailty scales and two physical performance tests:

#### Fried Frailty Phenotype

It defines frailty as a specific physical phenotype consisting of five possible components including unintentional weight loss, self-reported exhaustion, weakness, slowness and low physical activity [1]. The operational definition of each component is reported in Supplementary Appendix 1. The Fried Frailty Pheno-type (FFP) is one of the most commonly used frailty measures and it has been suggested by experts for frailty identification in hypertensive older adults [29]. The frailty phenotype is defined when three or more components are present [1].

#### Frailty Index

It defines frailty as a cumulative burden of health deficits, including symptoms, diseases, disabilities and other health impairments. It is expressed as a ratio of deficits present out of the total number of variables considered, providing a score on a continuum from 0 (no deficits) to a theoretical maximum of 1 (all items exhibit deficits) [30]. The Frailty Index methodology was applied in the two clinical trials that investigated frailty in older hypertensive adults [25,26]. The Italian version validated by Abete *et al*. [31] was applied in the present study (Supplementary Appendix 2), with a cut-off value of more than> 0.25 [32].

#### Clinical Frailty Scale

It scores frailty using a nine-point scale based on clinical judgment (as informed by a clinical assessment), with each point corresponding to a written description of frailty status complemented by a pictorial representation [33]. Individuals with a score of at least 4 (i.e. very mildly frail or above) were considered to be frail. The Clinical Frailty Scale (CFS) is brief and easy to perform in routine practice, and therefore, it has been suggested for use in hypertensive patients by some authors [24], although inter-rater reliability might be lower among nongeriatric specialists [34].

#### Frailty Postal Score

It is a six-item questionnaire designed for self-administration by older people (Supplementary Appendix 3). It was proven to be accurate in identifying FFP and predictive of adverse health outcomes in community-dwelling older people, with a cut-off score of at least 6.5 [35].

#### Gait speed (m/s)

It is measured on a 4-m walking distance, with use of a walking aid permitted if routinely used by the participant to walk short distances. Gait speed was shown to modify the association between BP and adverse outcomes [17,36] and can be easily measured in routine practice. Individuals with gait speed less than 0.8 m/s were considered to be frail in the present study [17,37].

#### Short Physical Performance Battery

It assesses lower-extremity function using three separate tests, that is standing balance, gait speed and repeated chair stands [38]. A summary performance score is created from the single tests, ranging from 0 to 12, with higher scores indicating better lower-extremity performance. It was found to be highly predictive of several outcomes of geriatric interest including disability, falls, hospitalization and mortality [39]. Individuals with a score of 8 or less were considered to be frail in the present study.

### Blood pressure and other measures

All participants underwent office BP measurements, with SBP and DBP measured twice in the sitting position after resting for 5 min. Orthostatic BP was assessed during 3-min active standing in participants who were able to stand and orthostatic hypotension was defined as a SBP fall at least 20 mmHg or to SBP less than 90 mmHg and/or a DBP fall at least 10 mmHg [40]. Twenty-four-hour ABPM was performed using a validated oscillometric device (TM-2430; A&D, Tokyo, Japan) with readings obtained automatically at 15-min intervals during the daytime and at 20-min intervals at night-time. Mean ambulatory daytime, night-time, and 24-h SBP and DBP were recorded and ‘white-coat’ effect was assessed, defined as a difference more than 20 mmHg between office SBP and mean ambulatory daytime SBP. Night-time BP dipping was measured as the difference between mean daytime and night-time SBP, expressed as percentage of daytime SBP. On the basis of nocturnal dipping, participants were classified as ‘dippers’ (preserved circadian rhythm with nocturnal dipping 10–20%), ‘nondippers’ (absence of circadian rhythm with nocturnal dipping between <10%), ‘reverse dippers’ (reversed circadian rhythm with a nocturnal rise in BP) and ‘extreme dippers’ (preserved circadian rhythm with nocturnal dipping >20%).

Disease burden was assessed using the Charlson Comorbidity Index. Functional status was assessed by the self-maintenance of the basic (BADLs, score 0–6) and instrumental (IADLs, score 0–8) activities of daily living [41,42], with disability defined as loss of autonomy in at least two basic and at least one instrumental activities. Depressive symptoms were investigated using the 15-item Geriatric Depression Scale, using the 6+ cut-off [43]. A dichotomic assessment of physical activity was performed, with a positive answer if aerobic physical activity (playing sport, walking, dancing, gardening, aerobics and so on) was reported at least once per week.

### Ethics

The HYPER-FRAIL project was carried out in compliance with the Declaration of Helsinki for Human Research. The study was approved by the Local Research Ethics Committee (protocol reference number: 16539_oss). Each participant or his/her legal representative gave written informed consent prior inclusion in the study.

### Statistical analysis

Data are presented as means with standard deviations (SD) for normally distributed continuous variables, as medians and interquartile ranges (IQRs, 25th to 75th percentiles) for nonnormally distributed variables and as absolute frequencies with percentages (*n*, %) for categorical variables. The independent samples *t*-test (parametric) or the Mann–Whitney *U* test (nonparametric) was used as appropriate for comparisons of continuous variables. For categorical variables, differences between groups were tested using the chi-square test.

Agreement between frailty instruments was examined with the Cohen kappa statistic [44]. Moreover, a Venn diagram was constructed (http://www.interactivenn.net/index.html) to determine the overlap between the four frailty scales used in the study (FFP, FI, CFS, FPS).

Multivariable binary logistic regression models were fitted independently for each frailty definition to identify clinical variables associated with frailty (‘correlates of frailty’), adjusting for demographics and clinical variables showing an association with the outcome in univariate analysis. Results of logistic regression were expressed as odds ratios (ORs) and 95% confidence intervals (95% CIs).

Statistical significance was set at a *P* value less than 0.05. All statistical analyses were performed using SPSS software version 26 (SPSS, Inc., Chicago, Illinois, USA).

## RESULTS

The study sample included 121 patients (mean age 81 years, age range 75–92 years); 60% were women. Clinical features, functional status and BP values of participants are detailed in Table 1.

**TABLE 1:** Characteristics of the study sample

	Study sample (*n* = 121)
Age (years), mean (SD)	81.2 (4.4)
Female/male, *n* (%)	72 (59.5) /49 (40.5)
Charlson Comorbidity Index, median (IQR)	5 (4–6)
Number of daily medications, median (IQR)	7 (5–9)
Number of antihypertensive medications, median (IQR)	2 (2–3)
Coronary artery disease, *n* (%)	19 (15.7)
Stroke/transient ischemic attack, *n* (%)	28 (23.1)
Diabetes, *n* (%)	27 (22.3)
Dyslipidaemia, *n* (%)	82 (67.8)
Chronic kidney disease, *n* (%)	75 (62.0)
Atrial fibrillation, *n* (%)	11 (9.1)
Heart failure, *n* (%)	6 (5.0)
Parkinson's disease, *n* (%)	5 (4.1)
Depressive symptoms, *n* (%)	34/119 (28.6)
Mild cognitive impairment, *n* (%)	11 (9.1)
Dementia, *n* (%)	36 (29.8)
History of falls during the previous year, *n* (%)	54 (44.6)
History of syncopal episodes during the previous year, *n* (%)	12 (9.9)
Disability in BADL, *n* (%)	22 (18.2)
Disability in IADL, *n* (%)	63 (52.1)
Living alone, *n* (%) Living with partner / family, *n* (%) Living with a caregiver, *n* (%)	27 (22.3) 86 (71.1) 8 (6.6)
Daily/weekly physical activity, *n* (%)	56 (46.3)
Use of a walking aid, *n* (%)	24 (19.8)
Office SBP/DBP, mean (SD)	152.9 (20.8) / 79.7 (13.2)
Orthostatic hypotension (*n* = 119), n (%)	51 (42.9)
Home SBP / DBP (*n* = 87), mean (SD)	137 (11.9) / 73.7 (8.4)
Daytime SBP / DBP (ABPM), mean (SD)	149.6 (15.3) / 79.1 (8.9)
24h SBP / DBP (ABPM), mean (SD)	147.1 (15.6) / 76.8 (8.8)
Night-time SBP / DBP (ABPM), mean (SD)	137.7 (20.9) / 68.7 (10.1)
Dipping profile (*n* = 117), n (%) Dipper Nondipper Reverse dipper Extreme dipper	35 (29.9) 51 (43.6) 19 (16.2) 12 (10.3)
White coat effect, *n* (%)	18 (14.9)

ABPM, ambulatory blood pressure monitoring; BADL, basic activities of daily living; IADL, instrumental activities of daily living; IQR, interquartile range; SD, standard deviation.

**FIGURE 1:**
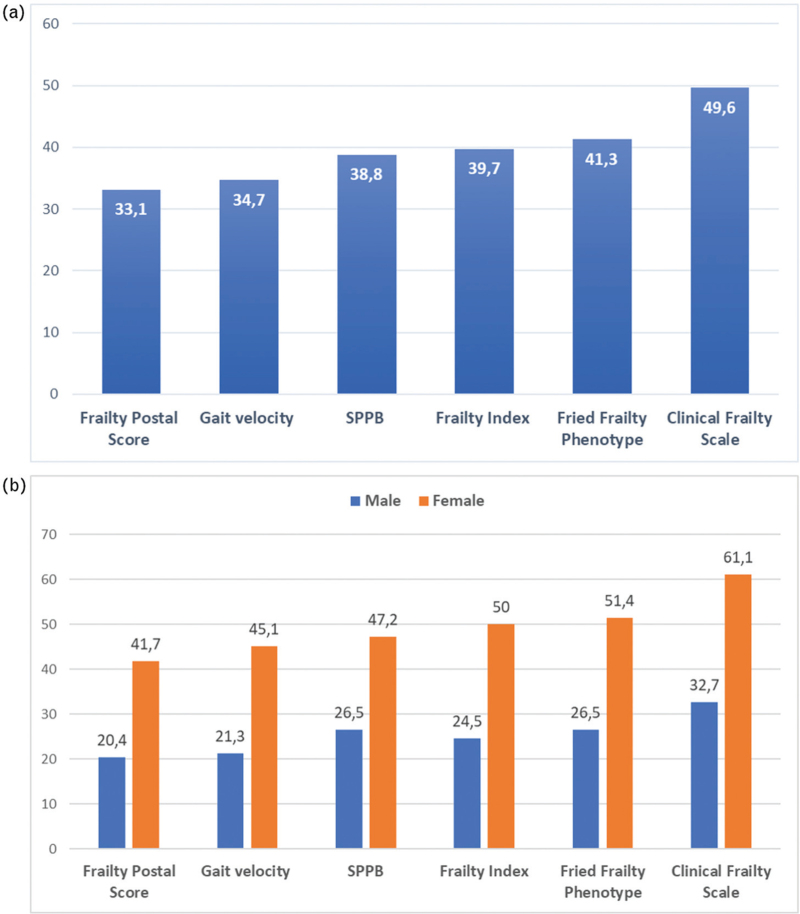
Prevalence of frailty according to different definitions: overall (a) and sex-specific prevalence proportions (b). Frailty Postal Score: *P* *=* 0.015; gait velocity: *P* *=* 0.008; Short Physical Performance Battery (SPPB): *P* *=* 0.022; Frailty Index: *P* *=* 0.005; Fried Frailty Phenotype: *P* *=* 0.006; Clinical Frailty Scale: *P* *=* 0.002.

The sample had a median of 2 (IQR 1–3) FFP components and a median CFS score of 3 (IQR 3–5). Median FI and median FPS were 0.12 (IQR 0.12–0.37) and 3.5 (IQR 1–7), respectively. The assessment of physical performance showed a mean gait speed of 0.84 (SD 0.3) m/s and a median SPPB score of 10 (IQR 7–11). The prevalence of frailty varied according to the definition used: the CFS scored the greatest proportion of patients as frail (50%), while the FPS scored the lowest one (33%). The FFP, FI and SPPB provided similar prevalence proportions, ranging between 39% and 41% (Fig. 1a). Frailty prevalence was higher in female than male participants, regardless of the definition used (*P* < 0.05 for all; Fig. 1b).

### Agreement between frailty definitions

**FIGURE 2:**
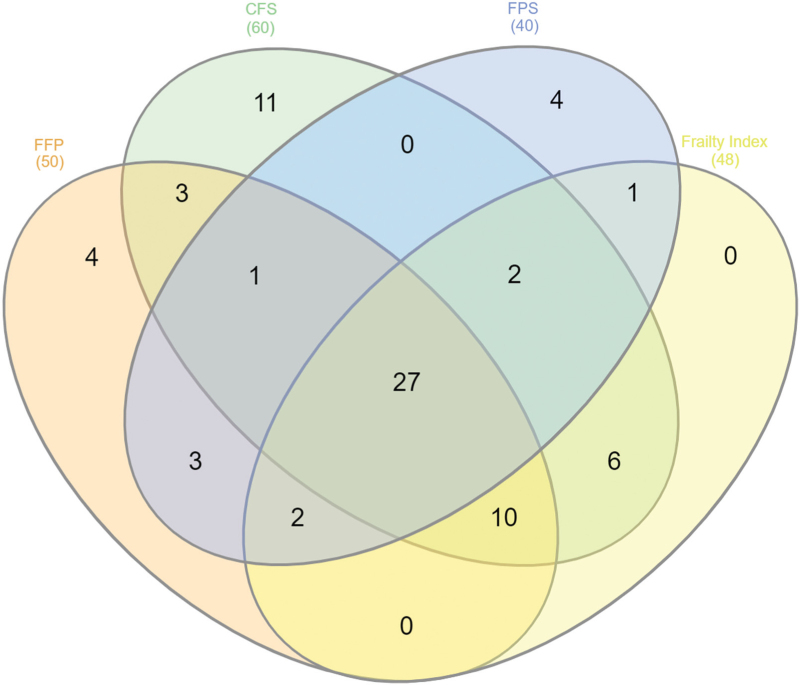
Venn diagram showing the overlaps between each frailty classification. The number of participants identified by each combination of instruments is shown by the number in each overlapping section. CFS, Clinical Frailty Scale; FFP, Fried Frailty Phenotype; FPS, Frailty Postal Score.

A Venn diagram showed relatively modest overlap between the four frailty scales applied in the study (Fig. 2). Indeed, among 74 participants classified as frail by any of the four frailty measures, 27 out of 74 (37%) were defined as frail according to all four definitions. Eleven participants were defined as frail only by the CFS and four were frail according to the FFP or the FPS only (Supplementary Table 1).

The Cohen's kappa coefficient indicated moderate agreement between FFP, FI and SPPB, and between FI and CFS (Kappa coefficient 0.60–0.79). Agreement was minimal between the CFS and the FPS (Kappa coefficient <0.40) and weak among the remaining measures (Kappa coefficient 0.40–0.59, Table 2).

**TABLE 2:** Agreement between frailty definitions according to Cohen's kappa coefficient

	Fried Frailty Phenotype	Frailty Index	Clinical Frailty Scale	Frailty Postal Score	Gait speed	SPPB
Fried Frailty Phenotype	–	0.657 (*P* < 0.001)	0.537 (*P* < 0.001)	0.579 (*P* < 0.001)	0.590 (*P* < 0.001)	0.605 (*P* < 0.001)
Frailty Index		–	0.702 (*P* < 0.001)	0.573 (*P* < 0.001)	0.514 (*P* < 0.001)	0.671 (*P* < 0.001)
Clinical Frailty Scale			–	0.337 (*P* < 0.001)	0.508 (*P* < 0.001)	0.553 (*P* < 0.001)
Frailty Postal Score				–	0.440 (*P* < 0.001)	0.517 (*P* < 0.001)
Gait speed					–	0.582 (*P* < 0.001)

Strong (K ≥0.80)	Moderate (K 0.60–0.79)	Weak (K 0.40–0.59)	Minimal (K < 0.40)

### Clinical correlates of frailty

Univariate analysis showed no significant association between frailty and office, home and ambulatory BP values, irrespective of how frailty was defined. Similarly, the prevalence of orthostatic hypotension and white-coat effect did not vary significantly by frailty status (Supplementary Table 2). Clinical variables associated with frailty in univariate analysis included age, female sex, comorbidity burden, dementia, depressive symptoms, disability and physical performance. Frail participants were older, had greater burden of comorbidities and daily medications, and had higher disability and worse physical performance than nonfrail participants. Moreover, cognitive impairment and depressive symptoms were more prevalent in frail participants. A comparison of frail and nonfrail participants according to FFP and gait speed is detailed in Supplementary Table 3. Similar differences were reported between frail and nonfrail participants when frailty was defined according to FI, FPS, CFS or SPPB (data not shown).

In the multivariable analysis (Table 3), the use of walking aids and the presence of depressive symptoms according to the Geriatric Depression Scale were independently associated with frailty, regardless of the definition used. Correlates of frailty also included dementia and IADLs disability, which were independently associated with frailty status according to FI, CFS and gait speed. The burden of comorbidities was independently associated with frailty as per FI and gait speed. Physical exercise was inversely associated with frailty when the latter was defined according to FFP, FI and FPS.

**TABLE 3:** Multivariable logistic regression to identify correlates of frailty (dependent variable) defined according to different frailty tools (odds ratio, 95% confidence intervals)

	Disability in IADL	Dementia	Charlson Index	Use of walking aids	Depressive symptoms	Daily/weekly activity
Fried Frailty Phenotype	2.03 (0.70–5.91)	1.64 (0.57–4.73)	1.05 (0.73–1.51)	10.23 (2.27–46.10)	4.64 (1.54–14.01)	0.26 (0.09–0.74)
Frailty Index	12.98 (2.85–59.03)	9.03 (2.56–31.87)	2.18 (1.22–3.91)	49.93 (4.29–581.06)	6.39 (1.39–29.42)	0.13 (0.03–0.56)
Clinical Frailty Scale	12.48 (3.78–41.20)	12.43 (3.50–44.03)	1.49 (0.99–2.24)	6.99 (1.15–42.41)	7.10 (1.80–27.90)	1.85 (0.59–5.80)
Frailty Postal score	1.26 (0.41–3.89)	1.22 (0.41–3.68)	0.97 (0.68–1.38)	6.73 (1.76–25.79)	5.65 (1.93–16.55)	0.19 (0.06–0.59)
Short Physical Performance Battery	2.69 (0.93–7.83)	2.46 (0.87–6.96)	1.19 (0.84–1.68)	15.31 (3.05–76.79)	4.69 (1.53–14.39)	1.49 (0.52–4.24)
Gait speed	4.68 (1.42–15.41)	4.31 (1.46–12.74)	1.58 (1.05–2.39)	8.68 (1.93–38.99)	4.16 (1.18–14.59)	0.58 (0.19–1.78)

Variables included in the model: age, sex, disability in Instrumental Activities of Daily Living (IADL), depressive symptoms at the Geriatric Depression Scale (score >5), use of walking aids, regular physical activity, living alone, Charlson Comorbidity Index (not age-adjusted). Odds of dementia were adjusted for age, sex, depression diagnosis, use of walking aids, regular physical activity, living alone.

## DISCUSSION

The present study investigated frailty in a sample of hypertensive older outpatients, applying six commonly utilized frailty identification tools. The main results can be summarized as follows: frailty and hypertension frequently coexist in older outpatients, with at least one in three hypertensive older adults being labelled as ‘frail’ in the studied sample, independently of the frailty measure used; however, the prevalence of frailty varied substantially across the different tools and there was a modest overlap between the four frailty scales; 24-h BP values were not associated with frailty, however defined; and the use of walking aids and the presence of depressive symptoms were the only variables significantly associated with all frailty tools.

To date, few studies have analysed the prevalence of frailty in older patients with hypertension, providing highly variable proportions ranging from 3 to 68% according to the clinical setting and the adopted assessment tool [9,45,46]. In older adults participating in the HYVET and SPRINT studies, frailty status was identified using the FI and a left skewed distribution of scores was observed, with median values of 0.17 and 0.18, respectively [12,25,26]. Among SPRINT participants aged 75 years or older, prevalence of frailty according to the Frailty Index was 30.9% [25]. In the present study, the prevalence of frailty according to the Frailty Index was 40%, even though a higher Frailty Index cut-off (>0.25) was applied compared with that used in the SPRINT study (>0.21). A similar left skewed distribution of Frailty Index scores was described in our sample, but the median Frailty Index value (0.21, IQR 0.12–0.37) was higher than that reported in the SPRINT and HYVET samples, thus highlighting a discrepancy between data derived from clinical trials and the ‘real world’ and raising questions regarding the generalizability of evidence arising from those studies. Indeed, the prevalence of frailty in the HYVET and SPRINT studies was lower than that observed in other clinical studies [32,47] and both trials included participants with mild-to-moderate frailty [48], while individuals with the highest levels of frailty (e.g. with previous stroke or dementia and nursing home residents) were excluded.

The proportion of frailty observed in the current study is also higher than what previously reported in community-dwelling samples. In a systematic review of cross-sectional data from community-dwelling adults, the overall weighted prevalence of frailty was 10.7% [49]. In a meta-analysis of cross-sectional studies, the mean prevalence of frailty among individuals with hypertension was estimated to be 14% (95% CI 12--17) [9]. However, community samples typically include relatively healthy and active participants, with lower mean age and higher levels of functional autonomy and physical performance compared to clinical samples [49–52]. The HYPER-FRAIL pilot study included participants with greater comorbidity burden and higher clinical complexity as compared to a general community sample, and our observed prevalence is consistent with the 37% prevalence of Fried Frailty Phenotype observed in a sample of older individuals with hypertension from an outpatient cardiology clinic [53].

In the HYPER-FRAIL pilot study, frailty prevalence varied depending on the frailty definition used, ranging from 33 to 50%. The wide range of prevalence rates described in the present study might be attributable to the diversity in the components and development background of each frailty scale, leading to a gap between selected frail populations from each instrument. Consistently, previous research studies conducted in different clinical settings confirmed that different operationalizations of frailty result in widely differing prevalences [49,54–56]. In the Health and Retirement Study, a modified frailty phenotype identified 11% of older adults as frail, while the FI identified 32% [54]. A similar heterogeneity was described in a secondary analysis of the SHARE study, wherein eight frailty scales were compared reporting widely different estimates for the prevalence of frailty [55]. Finally, in a cross-sectional study carried out at a geriatric outpatient clinic in Canada, the prevalence of frailty was 35% according to the Fried's Frailty Phenotype and 50% according to the SPPB (≤9) [56].

The HYPER-FRAIL pilot study confirms the limited agreement between frailty identification tools, as revealed by the Venn diagram and the kappa statistics. These results support previous studies showing that different frailty scales capture different although partially overlapping groups of older adults. In the Tree-City study, the concordance between Fried's criteria, the Frailty Index and the Tilburg Frailty Indicator was very low, with only 15% of participants being identified as frail by all the scales [52]. Similarly, in a recent prospective observational study of older adults from four U.S. communities, 12% of individuals were deemed frail by both Fried's criteria and the Frailty Index [57]. The generally poor agreement between frailty measures may not be unexpected, as they are based on different theoretical constructs of frailty. It might be hypothesized that different frailty tools capture decline in different – although overlapping – health domains [51,57]. Frailty thus appears as a heterogeneous condition including different subtypes of physiological impairments that can have different weights when the identification is performed with different frailty tools. Consistently, the present study observed common clinical characteristics associated with frailty status, however defined, although independent clinical predictors partly differ for different tools. In particular, walking aids and depressive symptoms emerged as frailty markers, regardless of the definition used. Walking aids could represent an epiphenomenon or a proxy of multiple conditions that are known to be related to frailty, such as sarcopenia, poor physical performance, multimorbidity, disability and inactivity [54,58]. As regards depressive symptoms, previous literature has consistently reported a similar association with frailty [59,60], including in hypertensive patients [61]. It has been earlier suggested that this association might be related to shared clinical characteristics, such as weight loss, low level of physical activity and exhaustion [60]. By contrast, some authors hypothesise a more complex and two-way causal link. In the present study, the association does not seem to be explained by associated comorbidities, as suggested by other studies [62]. Yet, depressive symptoms may also represent the early manifestation of frailty, worsening mood in the context of a multisystem decline.

No significant association of frailty with office, home and ambulatory BP was detected in this sample, suggesting that BP values probably show a weaker association with frailty in older hypertensive adults in comparison with other clinical variables. Indeed, some previous studies reported similar results, showing no cross-sectional association between BP and frailty status, particularly at advanced age [46,63]. However, it cannot be excluded that our findings might have been influenced by the limited sample size. A number of literature studies has evaluated the association between frailty and BP and conflicting results have been described [9]. Some authors reported a higher prevalence of hypertension and poor BP control in frail individuals, particularly on ABPM [8,46,64], while some others described an association between frailty and low BP [32,65]. Finally, some studies indicate a more complex, U-shaped association between frailty and BP, implying that frail older adults may present with both low and high BP values [8,66]. Further and larger studies adopting different frailty definitions are thus needed to identify possible BP profiles of frail hypertensive subjects.

Other frailty correlates were identified in this study, including disability in instrumental ADLs, dementia and comorbidity burden. Consistently, multiple studies have shown cross-sectional and prospective associations of frailty with cognitive impairment with possible bidirectional causal links [61,67]. It is also clear from the literature that a significant overlap exists between frailty and disability and that frailty does not simply represent a predisability stage [54,58,68]. Indeed, prevalence of disability has been reported to rise with increasing Frailty Index. Also, frailty and multimorbidity commonly co-occur, as do multimorbidity and disability [54,68]. Therefore, even though frailty is distinguishable from multimorbidity and disability, all three concepts are closely related and may contribute to each other.

The analysis of frailty correlates seems to confirm that different frailty instruments identify partly different frailty profiles. Indeed, dementia, disability and comorbidities were associated with frailty when defined according to the cumulative deficit model, while association with measures of physical frailty, that is, the Fried's model and physical performance measures, was milder or absent. The Frailty Index was strongly associated with most variables tested and emerged in this study as a measure of clinical complexity. At odds with the FFP, both the Frailty Index and the CFS do not identify frailty as a single physical domain within a CGA, but rather provide a multidimensional evaluation of health status resulting from the interplay of disease burden, disability, cognitive and sensory impairment, and psycho-social variables (the Frailty Index) or from a trained and expert global clinical impression based on a geriatric evaluation (the CFS). Moreover, although most FFP models simply recognize the presence or absence of frailty (and, in some cases, prefrailty) status, health-deficit scales such as the CFS and the Frailty Index may be used as continuous scales, thereby recognizing increasing severity levels of frailty, which might have relevant implications in clinical-decision making.

In view of these differences, it is probably not appropriate to consider these tools as alternatives or interchangeable, but it is rather reasonable to apply different tools based on the specific requirements of each clinical setting [3,69].

### Limitations and Strengths

Some limitations of this study should be acknowledged. First, this is a pilot study involving a relatively small sample from a geriatric outpatient clinic, which may not be representative of older persons from other clinical settings and of the general community-dwelling population. In particular, frailty prevalence might have been overestimated due to the limited sample size and the specific geriatric setting. Moreover, the limited sample size might have hidden potential associations between frailty and BP values. Second, the cross-sectional design of the study did not allow for drawing conclusions on the timing sequence and causality of the relationship of frailty with its correlates. Third, a large number of frailty tools exists, which was not applied in this sample. Similarly, the present study did not consider grip strength (the weakness criterion of the FFP was fulfilled by chair stand test >15 s, Appendix 1), which was found to modulate the association between BP and adverse outcomes in previous research [70]. However, grip strength devices might not be available for routine assessment in nongeriatric settings.

The strength of this study lies in the comprehensive evaluation of participants using a relevant number of frailty instruments in the same cohort, which allowed for a more nuanced investigation of the overlapping prevalences of frailty in older patients with hypertension, using different frailty identification tools. Moreover, the comprehensive geriatric assessment allowed adjustment for a wide range of important confounders resulting in a comprehensive analysis of frailty correlates.

In conclusion, frailty is common among older outpatients with hypertension, but agreement among different assessment tools is weak-to-moderate, suggesting distinct albeit overlapping frailty profiles. Such divergent conceptualizations of ‘frailty’ might generate confusion among inexperienced users and inconclusive findings between studies. When managing frailty in the cardiovascular setting, a ‘gold standard’ assessment has not yet been identified. Therefore, large longitudinal studies are needed to assess the clinical implications of different frailty tools in predicting the prognosis of hypertensive older individuals and, possibly, their differential impact on the choice of antihypertensive treatments.

## ACKNOWLEDGEMENTS

The study was supported by Fondazione Cassa di Risparmio di Firenze.

### Conflicts of interest

The authors have no conflicts of interest to declare.

## Supplementary Material
