# Supplementary material for: Prevalence and correlates of frailty in older hypertensive outpatients according to different tools: the HYPER-FRAIL pilot study
Source: J Hypertens. 2023 Oct 3;42(1):86–94. doi: 10.1097/HJH.0000000000003559 (PMC10713004; doi:10.1097/HJH.0000000000003559)
Supplement: Supplementary file 1 [file jhype-42-086-s001.docx]

**Appendix 1.** Fried Frailty Phenotype.

| Frailty component | Operational definition |
| --- | --- |
| Unintentional weight loss | Self-reported or measured unintentional weight loss ≥5% in the past year |
| Self-reported exhaustion | Effort required for routine activities at least 3 days per week |
| Weakness | Low grip strength by chair stand test (>15 sec) |
| Slowness | Slow gait speed on a 4-meter walking distance (>4.7 sec) |
| Reduced physical activity | Moderate physical activity (playing sport, walking, dancing, gardening, aerobics …) at least 30 min/day 5 days per week |

**Appendix 2.** Modified Italian Frailty Index (English version)

| Need of help for personal hygiene activities | +1 (yes) |  |
| --- | --- | --- |
| Need of help for dressing | +1 (yes) |  |
| Need of help for getting up from chair | +1 (yes) |  |
| Need of help for home mobility | +1 (yes) |  |
| Need of help for feeding | +1 (yes) |  |
| Need of help for home care | +1 (yes) |  |
| Need of help for washing | +1 (yes) |  |
| Need of help for stair climbing | +1 (yes) |  |
| Need of help for weight lifting (4.5 kg) | +1 (yes) |  |
| Need of help for groceries | +1 (yes) |  |
| Need of help for home works | +1 (yes) |  |
| Need of help for meal preparation | +1 (yes) |  |
| Need of help for medical therapy management | +1 (yes) |  |
| Need of help for money management | +1 (yes) |  |
| Weight loss > 4.5 kg over the past year | +1 (yes) |  |
| Patient’s judgement of health status Poor / Modest  Good  Very good / Excellent | +1 / +0.75  +0.5  +0.25 / +0 |  |
| Health worsening over the past year | +1 (yes) |  |
| Lying in a bed >12h/day for health reasons over the past month | +1 (yes) |  |
| Activity restriction over the past month | +1 (yes) |  |
| Leaving the house <3 times per week | +1 |  |
| Exhaustion Often / Sometimes / Rarely | +1 / +0.5 / +0 |  |
| Geriatric depression scale >5 | +1 |  |
| Feeling sad Often / Sometimes / Rarely | +1 / +0.5 / +0 |  |
| Social support score > 13  6-13  < 6 | +1  +0.5  +0 |  |
| Movement difficulties Often / Sometimes / Rarely | +1 / +0.5 / +0 |  |
| Hypertension Yes / Possible | +1 / +0.5 |  |
| Angina (o coronary artery disease) Yes / Possible | +1 / +0.5 |  |
| Heart failure Yes / Possible | +1 / +0.5 |  |
| Stroke Yes / Possible | +1 / +0.5 |  |
| Neoplasia Yes / Possible | +1 / +0.5 |  |
| Diabetes Yes / Possible | +1 / +0.5 |  |
| Arthrosis Yes / Possible | +1 / +0.5 |  |
| Chronic obstructive pulmonary disease Yes / Possible | +1 / +0.5 |  |
| Dementia | + 1 |  |
| BMI < 18.5 or > 29  25-29  18.5-24.9 | +1  +0.5  +0 |  |
| Low muscle strength by chair stand test (>15 sec) | +1 |  |
| Gait speed (4 mt) >10 s / ≤10 s | +1 / + 0 |  |
| Malnutrition risk (MUST) low / moderate / high | +1 / +0.5 / +0 |  |

**Appendix 3.** Frailty Postal Score: items and relative weight.

| Item | Weighted score |
| --- | --- |
| Is your sight good enough to read newspaper heading, even with glasses? [No] | + 1.5 |
| Do you easily get exhausted in daily chores? [Yes] | + 3 |
| Do you have problems with your memory? [Yes] | + 1 |
| Did you have any falls in last 6 months? [Yes] | + 1.5 |
| Do you have difficulty walking 400 m on a flat surface? [Yes] | + 3.5 |
| Do you take 5+ drugs on a regular basis (daily or almost daily)? [Yes] | + 1 |
