# Supplementary material for: Prevalence and correlates of frailty in older hypertensive outpatients according to different tools: the HYPER-FRAIL pilot study
Source: J Hypertens. 2023 Oct 3;42(1):86–94. doi: 10.1097/HJH.0000000000003559 (PMC10713004; doi:10.1097/HJH.0000000000003559)
Supplement: Supplementary file 2 [file jhype-42-086-s002.docx]

**Supplementary Table 1.** Overlap among frailty instruments: number of participants identified by single instruments or their combination.

|  | N | Proportion (of 74 participants classified as frail by any of the four instruments) |
| --- | --- | --- |
| Participants classified as frail by a **single** instrument |  |  |
| Fried Frailty Phenotype | 4 | 5.4 |
| Frailty Index | 0 | 0 |
| Clinical Frailty Scale | 11 | 14.9 |
| Frailty Postal Score | 4 | 5.4 |
| Participants classified as frail by **two** instruments |  |  |
| Fried Frailty Phenotype – Frailty Index | 0 | 0 |
| Fried Frailty Phenotype – Clinical Frailty Scale | 3 | 4.1 |
| Fried Frailty Phenotype – Frailty Postal Score | 3 | 4.1 |
| Frailty Index – Clinical Frailty Scale | 6 | 8.1 |
| Frailty Index – Frailty Postal Score | 1 | 1.4 |
| Clinical Frailty Scale – Frailty Postal Score | 0 | 0 |
| Participants classified as frail by **three** instruments |  |  |
| Fried Frailty Phenotype – Frailty Index – Clinical Frailty Scale | 10 | 13.5 |
| Fried Frailty Phenotype – Frailty Index – Frailty Postal Score | 2 | 2.7 |
| Fried Frailty Phenotype – Frailty Postal Score – Clinical Frailty Scale | 1 | 1.4 |
| Frailty Index – Clinical Frailty Scale – Frailty Postal Score | 2 | 2.7 |
| Participants classified as frail by **four** instruments | 27 | 36.5 |

**Supplementary Table 2.** Office, home and ambulatory blood pressure values by frailty status.

|  | **Frailty Index** | | | | **Fried Frailty Phenotype** | | |
| --- | --- | --- | --- | --- | --- | --- | --- |
| Mean (SD) | **Frail** (n=48) | **Fit** (n=73) | **p** | | **Frail** (n=50) | **Non-frail** (n=71) | **p** |
| Office SBP | 151.5 (21.0) | 153.8 (20.8) | 0.561 | | 154.1 (21.7) | 152.0 (20.3) | 0.598 |
| Office DBP | 78.3 (13.2) | 80.6 (13.2) | 0.336 | | 80.4 (12.6) | 79.2 (13.7) | 0.648 |
| Home SBP (n=87) | 136.0 (11.5) | 137.7 (12.1) | 0.541 | | 136.7 (12.9) | 137.5 (11.3) | 0.752 |
| Home DBP (n=87) | 72.7 (10.8) | 74.2 (7.2) | 0.452 | | 73.9 (9.2) | 73.6 (7.9) | 0.876 |
| Daytime SBP* | 150.3 (13.1) | 149.2 (16.7) | 0.683 | | 149.8 (14.2) | 149.5 (16.1) | 0.937 |
| Daytime DBP* | 79.3 (10.5) | 79.0 (7.8) | 0.874 | | 79.0 (9.8) | 79.2 (8.3) | 0.893 |
| 24h SBP* | 147.8 (13.7) | 146.6 (16.8) | 0.687 | | 147.7 (14.6) | 146.6 (16.3) | 0.699 |
| 24h DBP* | 77.2 (10.5) | 76.5 (7.5) | 0.660 | | 76.6 (9.7) | 76.9 (8.1) | 0.874 |
| Night-time SBP* | 138.1 (20.3) | 137.4 (21.4) | 0.865 | | 140.1 (20.8) | 136.0 (21.0) | 0.296 |
| Night-time DBP* | 69.3 (11.6) | 68.3 (9.1) | 0.591 | | 69.8 (11.7) | 67.9 (8.9) | 0.321 |
| OH, n (%) | 24 (50.0) | 27 (38.0) | 0.195 | | 26 (52.0) | 25 (36.2) | 0.086 |
| White coat, n (%) | 7 (14.6) | 11 (15.1) | 0.942 | | 9 (18.0) | 9 (12.7) | 0.418 |
|  | **Clinical Frailty Scale** | | | | **Frailty Postal Score** | | |
| Mean (SD) | **Frail** (n=60) | **Fit** (n=59) | **p** | | **Frail** (n=40) | **Fit** (n=81) | **p** |
| Office SBP | 151.8 (21.7) | 154.0 (20.1) | 0.554 | | 152.5 (21.8) | 153.1 (20.5) | 0.880 |
| Office DBP | 78.7 (13.3) | 80.7 (13.1) | 0.402 | | 80.8 (12.0) | 79.2 (13.8) | 0.513 |
| Home SBP (n=87) | 138.0 (12.7) | 136.6 (11.3) | 0.590 | | 137.3 (14.3) | 137.1 (10.8) | 0.933 |
| Home DBP (n=87) | 74.3 (9.1) | 73.7 (8.0) | 0.619 | | 73.0 (10.0) | 74.1 (7.6) | 0.567 |
| Daytime SBP* | 151.1 (14.1) | 148.2 (16.4) | 0.301 | | 150.4 (16.1) | 149.3 (15.0) | 0.717 |
| Daytime DBP* | 79.6 (10.0) | 78.6 (7.8) | 0.323 | | 79.6 (10.5) | 78.9 (8.1) | 0.677 |
| 24h SBP * | 148.6 (14.4) | 145.6 (16.7) | 0.307 | | 147.7 (16.6) | 146.8 (15.2) | 0.770 |
| 24h DBP* | 77.6 (9.9) | 76.0 (7.5) | 0.563 | | 76.8 (10.5) | 76.7 (7.9) | 0.967 |
| Night-time SBP* | 138.9 (20.2) | 136.6 (21.7) | 0.542 | | 136.7 (23.2) | 138.2 (19.9) | 0.726 |
| Night-time DBP* | 69.8 (11.5) | 67.7 (8.6) | 0.250 | | 67.5 (10.8) | 69.3 (9.8) | 0.380 |
| OH, n (%) | 29 (48.3) | 22 (37.3) | 0.223 | | 19 (48.7) | 32 (40.0) | 0.367 |
| White coat, n (%) | 8 (13.3) | 10 (16.4) | 0.636 | | 5 (12.5) | 13 (16.0) | 0.606 |
|  | **Short Physical Performance Battery** | | | | **Gait speed** | | |
| Mean (SD) | **Score ≤8**  (n=47) | **Score >8**  (n=74) | | **p** | **<0.8 m/s**  (n=42) | **≥0.8 m/s**  (n=76) | **p** |
| Office SBP | 150.3 (22.6) | 154.6 (19.6) | | 0.270 | 149.7 (22.4) | 154.6 (20.0) | 0.232 |
| Office DBP | 77.5 (13.4) | 81.1 (13.0) | | 0.143 | 76.8 (12.7) | 81.7 (13.1) | 0.049 |
| Home SBP (n=87) | 137.3 (13.4) | 137.1 (11.2) | | 0.924 | 137.3 (13.4) | 136.6 (11.0) | 0.797 |
| Home DBP (n=87) | 73.0 (10.3) | 74.1 (7.4) | | 0.558 | 72.9 (10.1) | 73.9 (7.7) | 0.601 |
| Daytime SBP* | 150.8 (14.0) | 148.9 (16.1) | | 0.501 | 148.0 (14.3) | 150.1 (15.7) | 0.464 |
| Daytime DBP* | 78.9 (10.5) | 79.2 (7.9) | | 0.864 | 77.2 (10.0) | 80.0 (8.2) | 0.114 |
| 24h SBP* | 148.3 (14.7) | 146.3 (16.2) | | 0.504 | 145.5 (14.6) | 147.6 (16.0) | 0.490 |
| 24h DBP* | 76.9 (10.5) | 76.7 (7.6) | | 0.922 | 75.1 (9.6) | 77.6 (8.2) | 0.153 |
| Night-time SBP* | 138.5 (20.5) | 137.2 (21.3) | | 0.747 | 135.6 (19.2) | 138.4 (21.8) | 0.510 |
| Night-time DBP* | 68.8 (11.1) | 68.6 (9.6) | | 0.932 | 67.4 (10.1) | 69.3 (10.2) | 0.339 |
| OH, n (%) | 23 (48.9) | 28 (38.9) | | 0.279 | 22 (52.4) | 29 (39.2) | 0.169 |
| White coat, n (%) | 7 (14.9) | 11 (14.9) | | 0.997 | 7 (16.7) | 11 (14.5) | 0.751 |

**ABPM, ambulatory blood pressure monitoring. SD; standard deviation; DBP, diastolic blood pressure; SBP, systolic blood pressure; OH, orthostatic hypotension.*

**Supplementary Table 3**. Participants’ characteristics by frailty status as assessed using the Fried Frailty Phenotype (A) and gait speed (B).

| **A. Fried Frailty Phenotype** | | | |
| --- | --- | --- | --- |
|  | **Frail** (n=50) | **Non-frail** (n=71) | **p** |
| Age (years), mean (SD) | 82.3 (4.7) | 80.5 (4.1) | **0.028** |
| Female, n (%) | 37 (74.0) | 35 (49.3) | **0.006** |
| Charlson Comorbidity Index, median (IQR) | 5 (4-7) | 5 (4-5) | 0.066 |
| Nr. daily medications, median (IQR) | 8 (5-10) | 6 (5-8) | **0.002** |
| Coronary artery disease, n (%) | 9 (18.0) | 10 (14.1) | 0.560 |
| Stroke/Transient ischemic attack, n (%) | 14 (28.0) | 14 (19.7) | 0.287 |
| Atrial fibrillation, n (%) | 6 (12.0) | 5 (7.0) | 0.350 |
| Diabetes, n (%) | 11 (22.0) | 16 (22.5) | 0.944 |
| Chronic kidney disease, n (%) | 31 (62.0) | 44 (62.0) | 0.997 |
| Depressive symptoms (n=119), n (%) | 25/48 (52.1) | 9/71 (12.7) | **<0.001** |
| Mild Cognitive Impairment, n (%) | 4 (8.0) | 7 (9.9) | 0.726 |
| Dementia, n (%) | 22 (44.0) | 14 (19.7) | **0.004** |
| Disability in instrumental ADLs, n (%) | 37 (74.0) | 26 (36.6) | **<0.001** |
| Disability in basic ADLs, n (%) | 18 (36.0) | 4 (5.6) | **<0.001** |
| Living alone, n (%) | 11 (22.0) | 16 (22.5) | 0.944 |
| Daily/weekly physical activity, n (%) | 13 (26.0) | 43 (60.1) | **<0.001** |
| Walking aid, n (%) | 21 (42.0) | 3 (4.2) | **<0.001** |
| SPPB score, median (IQR) | 6.5 (3-9) | 11 (10-12) | **<0.001** |
| Gait speed, mean (SD) | 0.63 (0.27) | 0.98 (0.23) | **<0.001** |
| **B. Gait speed** | | | |
|  | **<0.8 m/s** (n=42) | **≥0.8 m/s** (n=76) | **P** |
| Age (years), mean (SD) | 82.9 (4.7) | 80.2 (3.9) | **0.001** |
| Female, n (%) | 32 (76.2) | 39 (51.3) | **0.008** |
| Charlson Comorbidity Index, median (IQR) | 5 (4-7.25) | 4 (3.25-5) | **<0.001** |
| Nr. daily medications, median (IQR) | 8 (5-10) | 6 (5-8) | **0.012** |
| Coronary artery disease, n (%) | 7 (16.7) | 12 (15.8) | 0.901 |
| Stroke/Transient ischemic attack, n (%) | 16 (38.1) | 12 (15.8) | **0.006** |
| Atrial fibrillation, n (%) | 6 (14.3) | 5 (6.6) | 0.168 |
| Diabetes, n (%) | 12 (28.6) | 15 (19.7) | 0.274 |
| Chronic kidney disease, n (%) | 27 (64.3) | 46 (60.5) | 0.687 |
| Depressive symptoms (n=119), n (%) | 21/41 (51.2) | 11/75 (14.7) | **<0.001** |
| Mild Cognitive Impairment, n (%) | 25 (59.5) | 21 (27.6) | **<0.001** |
| Dementia, n (%) | 23 (54.8) | 12 (15.8) | **<0.001** |
| Disability in instrumental ADLs, n (%) | 35 (83.3) | 26 (34.2) | **<0.001** |
| Disability in basic ADLs, n (%) | 16 (38.1) | 6 (7.9) | **<0.001** |
| Living alone, n (%) | 8 (19.0) | 18 (23.7) | 0.561 |
| Daily/weekly physical activity, n (%) | 13 (31.0) | 42 (55.3) | **0.011** |
| Walking aid, n (%) | 20 (47.6) | 4 (5.3) | **<0.001** |

*SD, standard deviation; IQR, interquartile range.*
